# Mucins and Pathogenic Mucin-Like Molecules Are Immunomodulators During Infection and Targets for Diagnostics and Vaccines

**DOI:** 10.3389/fchem.2019.00710

**Published:** 2019-10-22

**Authors:** Sandra Pinzón Martín, Peter H. Seeberger, Daniel Varón Silva

**Affiliations:** ^1^Department of Biomolecular Systems, Max Planck Institute of Colloids and Interfaces, Potsdam, Germany; ^2^Department of Biology, Chemistry and Pharmacy, Freie Universität Berlin, Berlin, Germany

**Keywords:** mucins, mucin-like molecules, O-glycoproteins, cancer, parasites, virus, infection

## Abstract

Mucins and mucin-like molecules are highly O-glycosylated proteins present on the cell surface of mammals and other organisms. These glycoproteins are highly diverse in the apoprotein and glycan cores and play a central role in many biological processes and diseases. Mucins are the most abundant macromolecules in mucus and are responsible for its biochemical and biophysical properties. Mucin-like molecules cover various protozoan parasites, fungi and viruses. In humans, modifications in mucin glycosylation are associated with tumors in epithelial tissue. These modifications allow the distinction between normal and abnormal cell conditions and represent important targets for vaccine development against some cancers. Mucins and mucin-like molecules derived from pathogens are potential diagnostic markers and targets for therapeutic agents. In this review, we summarize the distribution, structure, role as immunomodulators, and the correlation of human mucins with diseases and perform a comparative analysis of mucins with mucin-like molecules present in human pathogens. Furthermore, we review the methods to produce pathogenic and human mucins using chemical synthesis and expression systems. Finally, we present applications of mucin-like molecules in diagnosis and prevention of relevant human diseases.

## Introduction

Physical protection from external pathogens and molecules is essential for cell survival. Most mammal cells exposed to the external environment use complex molecular shields and coats that are present either as a hard shell (skin) or as a soft secretion (mucus) (Hansson, 2019). Mucus is present on the ocular surface and in organs of respiratory, gastrointestinal, and reproductive tracts. It covers human organs and glands and contains proteins having highly O-glycosylated repeats, called mucins (Corfield, 2015; Bansil and Turner, 2018).

Some human pathogens use similar protection mechanisms involving highly O-glycosylated proteins (Buscaglia et al., 2006). These molecules are present in parasites, viruses and fungi and include mucin-like regions (Herpes virus), mucin-like domains (Ebola virus and *Toxoplasma Gondii*), mucin-like glycoproteins (*Cryptosporidium parvum*), mucin-associated surface proteins MASPs (*Trypanosoma cruzi*), and mucin-type proteins (*Candida albicans*), among others. In this review, we use the term mucin-like molecules (MLMs) to denote all these molecules.

Mucins and MLMs share, as a structural feature, the presence of a dense array of O-linked oligosaccharides attached to serine or threonine residues of the protein. These glycans form a cover acting as a shield for protection and interaction with receptors (Buscaglia et al., 2006). A human mucin barrier protects the mucosal membranes and takes part in cellular regeneration, differentiation, signaling, adhesion, immune response, and tumor progression (Kufe, 2009; Senapati et al., 2010). Mucins and MLMs of protozoa, viruses and fungi protect these pathogens from the vector and vertebrate-host defense mechanisms and can have a critical role in targeting, attachment and invasion of specific host cells and tissues (Buscaglia et al., 2006; Lee and Saphire, 2009).

Comprehensive reviews about the structure, properties, role in cancer, and other aspects of mucins (Corfield, 2017; van Putten and Strijbis, 2017; Bansil and Turner, 2018; Dhanisha et al., 2018; Wagner et al., 2018; Kasprzak and Adamek, 2019) prompt us to cover these aspects only briefly by providing an overview of mucins and their comparison with MLMs. We will focus in the distribution, role in diseases and chemical structure of human mucins and pathogenic MLMs and review the role of these molecules as immunomodulators and their potential use in the diagnosis and prevention of diseases. Finally, we summarize the strategies required to obtain these complex molecules.

### Human Mucins

Mucus is a complex dilute aqueous viscoelastic secretion containing water, electrolytes, lipids, and proteins (Bansil and Turner, 2018). It is abundantly present in the epithelium of the gastrointestinal, respiratory and reproductive tracts and the secretory epithelial surfaces of liver, pancreas, gallbladder, kidney, and eyes, as well as in salivary and lacrimal glands. Mucus has diverse functions attributed to its primary structural component, mucins, which are present at concentrations between 1 and 5% (Rachagani et al., 2009; Corfield, 2015; Bansil and Turner, 2018). Mucins are expressed by epithelial cells (including endothelial cells), specialized epithelial cells known as goblet cells, leukocytes, and glands of the gastrointestinal tract (Tarp and Clausen, 2008; Rachagani et al., 2009; Dhanisha et al., 2018; Kasprzak and Adamek, 2019). They are present in the ocular surface and ear epithelium (Dhanisha et al., 2018) and cover the epithelial cell surfaces of the respiratory, digestive, and urogenital tracts forming gel-like structures (Johansson et al., 2008, 2014). Mucins form a protective barrier on the cell membrane and participate in regulation of solute transport, and as receptors for commensal and pathogenic microbes and for leukocyte targeting (Pelaseyed et al., 2014; Birchenough et al., 2015). Mucins are also associated with cellular regeneration, differentiation, integration, signaling, adhesion, and apoptosis (Bergstrom and Xia, 2013; Pelaseyed et al., 2014; Corfield, 2017; Kasprzak and Adamek, 2019).

Human mucins are encoded by 22 genes, designated MUC1 to MUC22, have a variable expression among tissues and display in the gastrointestinal tract the highest level and diversity (Behera et al., 2015). Mucins have a complex molecular organization and are classified into secreted and membrane bounded (transmembrane-) mucins considering their structure and localization (Kufe, 2009; Rachagani et al., 2009; Lang et al., 2016; Dhanisha et al., 2018). Secreted mucins form an extracellularly protective layer over the organs working as a barrier against external pathogens (Dhanisha et al., 2018). They can be gel-forming mucins (MUC 2, 5AC, 5B, 6, 19) or non-gel forming mucins (MUC 7, 8). MUC1, 3A, 3B, 4, 12, 13, 15, 16, 17, 18, 20, and 21 have a transmembrane domain attaching the glycoprotein to the membrane. Besides protection, they have a role in signaling, monitoring and repairing damaged epithelia (Martínez-Sáez et al., 2017; van Putten and Strijbis, 2017; Bansil and Turner, 2018; Dhanisha et al., 2018). Three mucins remain unclassified, the oviductal glycoprotein 1 (MUC9), endomucin MUC14 and MUC22 (Wagner et al., 2018).

Mucins contain variable glycosylated tandem repeat domains rich in proline (Pro), threonine (Thr) and/or serine (Ser) (PTS domains), and cysteine-rich regions localized at the amino and carboxy terminus and interspersed between the PTS domains (Bansil and Turner, 2018). The apomucin, or protein core, and the oligosaccharides are different among mucins (Corfield, 2015). An altered expression, up or down regulation, qualitative disturbances in glycosylation, changes in protein sequence, and in the structure of the glycans are generally associated with diseases, i.e., cancer (Brockhausen, 2003; Sheng et al., 2012; Nath and Mukherjee, 2014; Kasprzak and Adamek, 2019).

The expression of mucins was initially associated with epithelial tissues and later on with the immune system. This was particularly valid to MUC1 expressed by T and B cells (Agrawal et al., 1998; Chang et al., 2000; Treon et al., 2000; Correa et al., 2003; Fremd et al., 2016), MUC15 is expressed in adult human spleen, thymus, peripheral blood leukocyte, bone marrow, and lymph node (Pallesen et al., 2008), and MUC21 is expressed in thymus (Itoh et al., 2007). However, some mucins are found in other organs with certain specificity. Examples of these mucins are MUC14, a membrane bound mucin highly expressed in vascular tissues (dela Paz and D'Amore, 2009; Zuercher et al., 2012); MUC9, a non-gel-forming mucin, that is secreted by oviductal epithelial cells of the female reproductive tract (Slayden et al., 2018); and MUC3A and MUC3B have only been detected in the gastrointestinal tract and ear (Pratt et al., 2000; Sheng et al., 2012; Dhanisha et al., 2018; Kasprzak and Adamek, 2019). Other mucins genes such as MUC10 and MUC11 have not been identified in humans (Dhanisha et al., 2018). Detailed information about the distribution of human mucins has previously been reviewed (Behera et al., 2015; Dhanisha et al., 2018).

Membrane and secreted mucins have a high molecular weight (>200 kDa) and are composed of a long peptide chain with multiple O-linked glycans that correspond to more than 50% (w/w) of the glycoprotein. In mammals, the glycans are attached to the side chain of the serine or threonine via a N-acetylgalactosamine (GalNAc) that can be further elongated into different structures. The protein core is organized into two broadly distinct regions: a central region rich in Pro, Ser, and Thr residues containing multiple O-glycosylation and the carboxy- and amino-terminal non-repeat regions with low amounts of Ser/Thr and relatively few O-glycosylations. These non-repeat regions are generally rich in cysteine and contain N-glycans involved in the folding, oligomerization, and surface location of the proteins (Linden et al., 2008; Jonckheere et al., 2013; Martínez-Sáez et al., 2017; Bansil and Turner, 2018).

**Figure 1:**
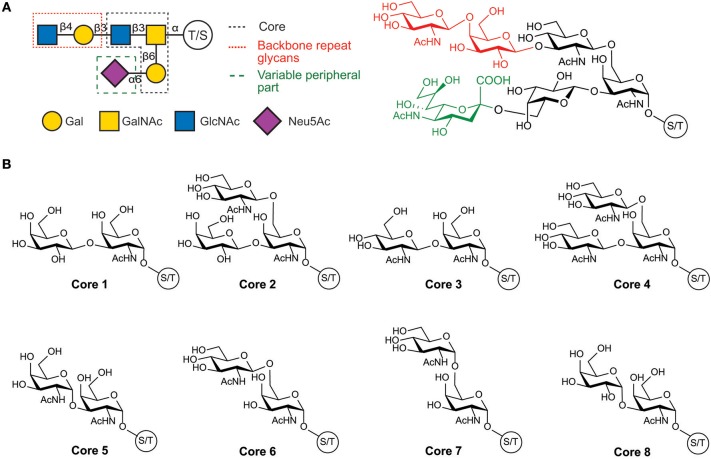
Structure of human mucin glycans. **(A)** Schematic and chemical representation of the organization of mucins glycans, **(B)** Structure of the glycan cores present in mucins.

The structure of the O-glycans present in human mucins comprises three main parts: the GalNAc linked to the protein; a backbone or extension part corresponding to an elongation of the GalNAc with either α-(1-3)- or β-(1-3)-linked galactose or by β-(1-6)-, β-(1-3)-, or α-(1-6)-linked N-acetylglucosamine; and a high variable peripheral part containing fucose and N-acetyl neuraminic acid terminal units. Glycan structures are summarized in Figure 1 and by Corfield (2015).

**Figure 2:**
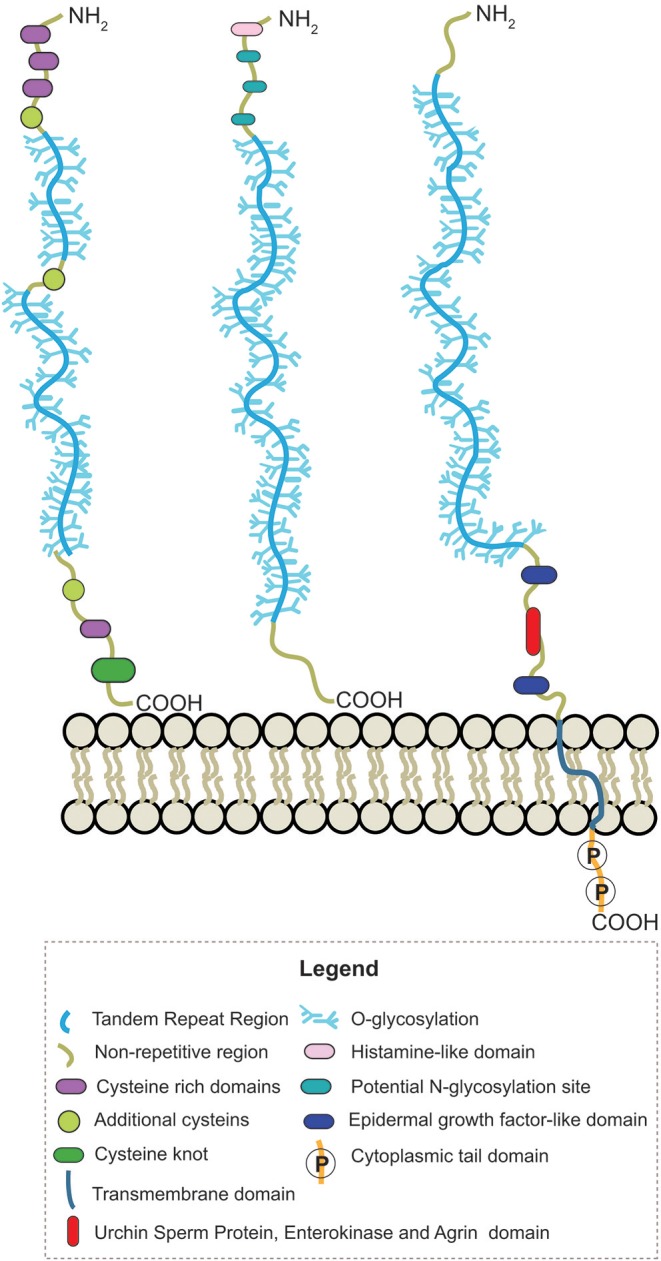
Schematic representation of the structure from secreted-gel-forming, secreted-non-gel forming and transmembrane human mucins.

Human mucin genes exhibit a specific domain called the variable number tandem repeat region (VNTR), encoding the tandem repeats region (TR) rich in PTS-domains and glycosylations. The presence of the PTS-domain is conserved in all mucins; however, the amino acid sequences and glycans within a mucin are identical but can vary among mucins. Secreted-gel-forming mucins have a TR with cysteine-rich regions flanked at its amino- and carboxy-terminus and interspersed between PTS domains (Bansil and Turner, 2018; Wagner et al., 2018). In addition, these mucins can also have von Willebrand-D-like-domains (VWF) flanking the amino and carboxy terminus of TR and cysteine knot (CK) at the carboxy terminus (Figure 2) (Ridley and Thornton, 2018). Differently, secreted non-gel-forming mucins only contain PTS and histamine-like domains. Membrane-bound-mucins have a common structure containing TRs, a transmembrane and a cytoplasmic tail domain (Xu et al., 2016; van Putten and Strijbis, 2017). Most of these mucins also contain Epidermal Growth Factor-like (EGF) and Sea Urchin Sperm Protein, Enterokinase, and Agrin (SEA) domains (Johansson et al., 2013; Jonckheere et al., 2013).

### Pathogenic Mucins

MLMs have been identified in the parasites *Trypanosoma cruzi* (Di Noia et al., 2002)*, Leishmania* (Ilg et al., 1999), *Toxoplasma gondii* (Tomita et al., 2018), *Cryptosporidium parvum* (Bhalchandra et al., 2013), and *Fasciola hepatica* (Noya et al., 2016). They are also present on the surface of the Ebola Virus (Lee et al., 2008), Herpes Simplex Virus (Altgärde et al., 2015) and in fungi, i.e., in *Candida albicans* (Altgärde et al., 2015). MLMs and mucins have similar functions acting as barrier to protect the membrane of the expressing cells (Buscaglia et al., 2006; Bergstrom and Xia, 2013), mediating interaction for cell penetration (Ricketson et al., 2015) or acting as signaling receptors in cells (van Putten and Strijbis, 2017).

Similar to human mucins, MLMs have domains rich in Pro, Thr and Ser containing multiple O-glycosylations. The structure of the glycan in MLMs from many pathogens is unknown, but some differences have been reported. Characterization of protozoan MLMs and *in vitro* studies showed important variations in the glycan core and the attachment of the glycans to Ser or Thr residues in *T. cruzi* MLMs via an N-acetylglucosamine (Previato et al., 1995).

**Figure 3:**
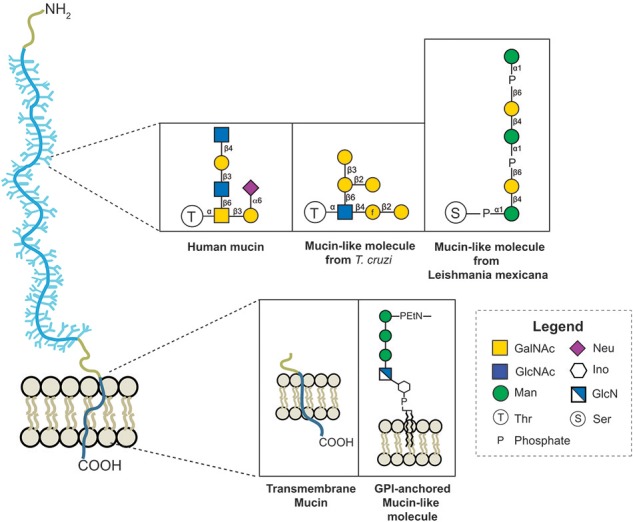
Variation of the protein-glycan and membrane linkage and glycan structures in human mucins and protozoan MLMs.

In some Leishmania MLMs, oligosaccharides are linked to proteins by a phosphodiester bond between the carbohydrate and Ser or Thr (see Figure 3) (Ilg et al., 1996; Ilg, 2000; Jain et al., 2001).

Besides Protozoa, trematode parasites also express MLMs that protect them from the host immune system and mediate their interaction with the host cells (Buscaglia et al., 2006; Wanyiri and Ward, 2006; Bhalchandra et al., 2013; Cancela et al., 2015). Characterization of cDNAs of proteins in *Fasciola hepatica* showed as particularities of these glycoproteins the presence of repeat Ser/Thr rich motifs with different lengths, minor amino acid variation and the absence of hydrophobic amino acids. The parasite *Cryptosporidium parvum* also express a MLM, *Cp*Clec, a type 1 transmembrane glycoprotein containing a canonical C-type lectin domain (CTDL), a signature long loop region hydrophobic core, a WIGL motif and highly O-glycosylated Ser-/Thr-rich domains (Bhalchandra et al., 2013). This composition suggests a role in attachment and invasion of host cells (Bouzid et al., 2013).

The protozoa *T. gondii* contains ML-domains in different surface related sequence proteins (SRS) that attach the parasite to the mammalian host cells and induce immune subversion during the acute infection. CST1, a key structural component of *T. gondii* cyst, is a glycoprotein conferring the sturdiness critical for persistence of bradyzoite forms (Tomita et al., 2013). CST1 contains 13 SRS domains and a stretch region with multiple Thr-rich tandem repeats that are similar to mucin-like domains observed in *C. parvum*. Recently, a similar 169 amino acid long stretch domain containing Thr-rich tandem repeats was determined in the SRS13 cyst wall protein between two SRS domains. These domains in SRS13 and CST1 cyst wall protein provide a physical barrier against proteolytic enzymes and may help to maintain the identity and hydration of the parasite (Tomita et al., 2018).

*Leishmania* parasites contain highly glycosylated MLMs with unique structural features, so-called proteophosphoglycans PPGs. These proteins contain phosphoglycosylation, Manα1-PO_4_-Ser, as a unique linkage between protein and glycan (Ilg et al., 1994, 1996; Moss et al., 1999). PPGs are secreted in the surface of the parasite and along with the lipophosphoglycan (LPG) form a dense matrix of filaments, so called filamentous PPG (fPPG), that surround the parasites and promote Leishmaniasis (Rogers et al., 2004; Rogers, 2012). A characterization of fPPG stablished that mostly phosphoglycans are present in the filaments (~96%). However, a small amount of amino acids (~4%) is also observed, and from them more than half of the amino acids are Ser and a large proportion of Ala or Pro. Most of the Ser residues are phosphoglycosylated (Ilg et al., 1999; Ilg, 2000).

The surface of the protozoan parasite *T. cruzi* is covered with MLMs and GPI-anchored glycoconjugates, termed mucins and mucin-associated surface proteins (MASP) (El-Sayed et al., 2005). *T. cruzi* mucins contribute to parasite protection and to establish a persistent infection (Buscaglia et al., 2006). These mucins have been extensively studied and encoded in two gene families: TcMUC encoding mucins in the mammalian stage and TcSMUG encoding mucins in the insect stages (Di Noia et al., 1998; Pech-Canul et al., 2017). These mucins share a common structure with three domains: a N-terminal SP, a central region showing high content (60–80%) of Thr, Ser, Pro, Gly, and Ala residues and a C-terminal signal for glycosylphosphatidylinositol (GPI) anchoring. The central region, present in the mature form of the proteins, bears multiple O-glycosylation sites and in some cases, a few (1-3) *N*-glycans (Cánepa et al., 2012b).

Early reports describing particular features of MLMs glycans derived from *T. cruzi* determined the linkage of glycans to threonine or serine *via* N-acetylglucosamine (Previato et al., 1994), and the abundance of GPI-anchored mucins in trypomastigotes (tGPI) containing glycans with terminal, non-reducing α-galactose (α-Gal) residues (Almeida et al., 1991). This α-Gal is part of the highly immunogenic epitope Galα(1,3)Galβ(1,4)GlcNAcα present on a mucin-like GPI-anchored glycoprotein present in sera of patients with chronic Chagas' disease that is recognized by anti-α-Gal antibodies (Almeida et al., 1993). This glycoprotein has a high carbohydrate content (60%), substantial amounts of Thr, Ser, Glu, Gly, Ala, Pro, *myo*-inositol, ethanolamine, and 1-O-hexadecylglycerol (Almeida et al., 1994).

Besides morphological variations in the life cycle of *T. cruzi*, there are important changes in the structure of glycolipids, GPIs attaching MLMs and carbohydrates characterizing the different stages of the parasite (de Lederkremer and Agusti, 2009). These changes include, among others, a higher content of GIPLs in epimastigotes than in trypomastigotes (Golgher et al., 1993; Pereira-Chioccola et al., 2000) and a change in the lipid part of GPILs from epismatigotes during the exponential and stationary growth phases from 1-*O*-hexadecyl-2-*O-*hexadecanoylglycerol to ceramide (de Lederkremer et al., 1993). Variations on the GPIs attaching MLMs include the lack of galactofuranose (Gal*f*) in the GPI-glycan of epimastigotes and trypomastigotes and a lipid change in trypomastigotes, which contain an alkylacylglycerol having mainly oleic and linoleic acid (Acosta Serrano et al., 1995; Previato et al., 1995; Almeida et al., 2000).

**Figure 4:**
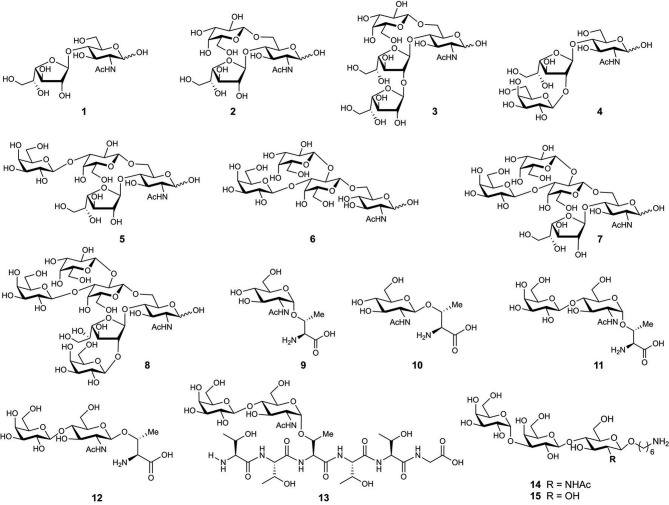
Chemical structure of synthesized glycans from MLMs of *Trypanosoma cruzi* Colombiana strain (**1**–**8**) and Y strain (**9**–**13**).

The first O-glycan characterized from *T. cruzi* MLMs showed oligosaccharide chains containing between three and six monosaccharide units that are conserved between *epimastigotes* and metacyclic *trypomastigotes* (Acosta Serrano et al., 1995). However, binding of anti-glycan antibodies showed the presence of the αGal(1,3)Gal epitope only in mucins from mammals, indicating a difference in mucins' glycosylation between mammals and insects (Almeida et al., 1994). In addition, there is polymorphism among the strains, the main difference being the presence of galactofuranose in glycans of the strains belonging to lineage I which includes G, Colombiana, and tulahuen (Figure 4) (Previato et al., 1994, 1995; Agrellos et al., 2003; Jones et al., 2004; Todeschini et al., 2009). Of particular interest is the *O*-glycans from mucins of *T. cruzi* from the Colombiana strain, due to the resistance of this strain to drugs used in Chagas' disease treatment. This strain, similar to the G-strain, presents a β-galactofuranose residue attached to N-acetylglucosamine (Todeschini et al., 2009). Additional glycosylated antigens described in *T. cruzi* may include a small surface antigen expressed in *trypomastigotes* (TSSA), which provides the first immunological marker to allow discrimination between lineages (Di Noia et al., 2002). Sequence analysis of TSSA showed high content of Ser and Thr residues in the protein backbone and multiple signals for putative O-glycosylation, suggesting that the gene encodes for a *T.cruzi* MLM (Di Noia et al., 2002). Further studies showed that TSSA play a role in host immune evasion, in maintaining the infection (Buscaglia et al., 2006) and in T. *cruzi* infectivity (Cánepa et al., 2012a). Contrary to initial studies suggesting that TSSA is glycosylated (Di Noia et al., 2002), a recent report described TSSA as a hypo-glycosylated molecule (Camara et al., 2017). Therefore, further research is still required to fully elucidate the TSSA structure and the presence of glycans.

An important group of MLMs are the viral mucin-like regions (MLRs). They are pathogenic factors in the Ebola virus (EBOV), Herpes Simplex Virus (HSV), Margburg virus (MARV), Crimean-Congo hemorrhagic fever virus (CCHFV), and human respiratory syncytial virus (hRSV) (Wertheim and Worobey, 2009). These regions should stretch the proteins to enhance their availability for binding, protecting the protein against proteolytic degradation, and acting as modulators of the host immune response (Wertheim and Worobey, 2009). EBOV has an envelope of glycoproteins that are crucial factors in determining virulence, including the MLR, called GP1. This highly glycosylated motif has N- and O-glycans (Kiley, 1988; Groseth et al., 2012) and has a similar structure to the HSV MLR (Altgärde et al., 2015). GP1 is essential for the infectivity of Zaire Ebola virus (ZEBOV) (Yang et al., 2000), and for the attachment of EBOV to host cells *via* interaction with surface lectins of hepatocytes, dendritic cells, macrophages, and endothelial cells (Fujihira et al., 2018). In HSV infections, a similar region from the gC glycoprotein balances the interaction and facilitate the attachment of viral particles to cells allowing an efficient release of viral progeny from the surface of infected cells (Altgärde et al., 2015).

MLMS are also present in fungi, with the Msb2 glycoprotein of *Candida albicans* as a main example. This high molecular weight and heavily glycosylated transmembrane protein is a sensor protein that takes part in the biosynthesis of the cell wall and in the invasion of solid surfaces (Whiteway and Oberholzer, 2004; Román et al., 2009; Szafranski-Schneider et al., 2012; Puri et al., 2015). Msb2 also protects *C. albicans* against antimicrobial peptides and can release its extracellular domain through a proteolytic cleavage generating a mucous layer to protect the cell. This protein is considered a functional analog of mammalian MUC1/MUC2 (Szafranski-Schneider et al., 2012).

## Mucins, MLMs, and Diseases

Modifications in mucins are strongly associated with diseases, susceptibility to pathogens, and the diagnosis and prognosis of cancer (Kasprzak and Adamek, 2019). An altered expression, up or down regulation of mucins, disturbances in glycosylation, and changes in the protein structure of mucins occur in many types of cancer (Rachagani et al., 2009; Hasnain et al., 2013; Nath and Mukherjee, 2014), inflammatory bowel disease, ocular surface diseases, and ulcerative colitis, among others (Dhanisha et al., 2018). Similarly, modification of MLMs protect pathogens from host proteases and recognition by the immune system, contributing to several infections (Ricketson et al., 2015; Noya et al., 2016; van Putten and Strijbis, 2017).

Cancer is a major global public health problem (Siegel et al., 2019) and its burden rose to 18.1 million new cases and 9.6 million cancer deaths in 2018 (Bray et al., 2018). In recent years, correlation studies showed an association between mucin overexpression and glycosylation with cancer formation, prognosis, and metastasis (Behera et al., 2015). MUC 1, 2, 3, 5AC, 5B, 8, 16, and 21 are related, to a different degree, in breast (Masaki et al., 1999), ovarian (Yin and Lloyd, 2001; Wang and El-Bahrawy, 2015), endometrial tumors (Hebbar et al., 2005), prostate (Xiong et al., 2006), pancreatic (Levi et al., 2004), gastric and cervical (Kaur et al., 2013), colorectal (Chang et al., 1994), renal cell carcinoma (Leroy et al., 2002), pseudoxyoma periotonei (Ciriza et al., 2000), and recently studied lung cancer (Yoshimoto et al., 2019). There are multiple recent reviews about the role on mucins and cancer for further reading (Chugh et al., 2015; Dhanisha et al., 2018; Kasprzak and Adamek, 2019).

In addition to cancer, mucins are also involved in other human diseases that commonly affect populations like asthma and otitis. Mucins 2, 5AC, 5B, and 6 are associated with diseases in epithelial tissue such as cystic fibrosis (Li et al., 1997; Puchelle et al., 2002; Thornton et al., 2008), MUC 3, 4, and 5AC in cap polyposis (Buisine et al., 1998), MUC19 in Sjörgen syndrome (Yu et al., 2008), and MUC 5B in diffuse panbronchiolitis (Kamio et al., 2005). Specific conditions in the eye and ear are also associated with mucins. Ethmoid chronic sinusitis is associated with MUC 4, 5AC, 5B, 7, and 8 (Jung et al., 2000), asthma with MUC 5AC and 7 (Watson et al., 2009) and chronic otitis media with MUC 4, 5AC, and 5B (Moon et al., 2000; Lin et al., 2001). Particular changes in mucins in diseases have been reported (Behera et al., 2015; van Putten and Strijbis, 2017; Kasprzak and Adamek, 2019).

In contrast to human mucins, little is known about the role of MLMs in infections. MLMs protect the pathogens (Puri et al., 2015) and ensure the targeting and invasion of specific cells or tissues (Buscaglia et al., 2006). Human secreted gel-forming mucins coat and protect mucosal surfaces from chemical, enzymatic, and mechanical damages (Portal et al., 2017) and from penetration and pathogen invasion. MLMs from pathogens may have similar functions; however, more studies are necessary to determine the mechanisms involving these molecules in pathogen protection from the host defense and in the degradation of protective mucus gels of the host.

Changes in sialylation levels in glycolipids and glycoproteins are a hallmark of human diseases (Amon et al., 2014). Nonetheless, this modification of glycans is also used by pathogens to improve their survival and pathogenicity. *T. cruzi* uses sialylation of proteins to avoid lysis by serum factors and to enhance the interaction with the host cells (Tomlinson et al., 1994). The parasites do not synthesize sialic acid (Jain et al., 2001), however, the mucins of the parasite membrane are acceptors for sialic acid that is transferred from the host proteins using trans-sialidases (Giorgi and de Lederkremer, 2011). Sialylation may also reduce the susceptibility of the parasite to anti-α-Gal antibodies present in the mammalian bloodstream (Pereira-Chioccola et al., 2000), allowing colonization and infection. Recently, *T. cruzi* mucins were also associated with parasite attachment to the internal cuticle of the triatomine rectal ampoule, a critical step leading to *T. cruzi* differentiation into infective forms to mammalian host cells (Cámara et al., 2019).

Proteophosphoglycans (PPG) from Leishmania parasites have different roles during infection. They contribute to binding of *Leishmania major* promastigotes and the survival of the parasites within the macrophages (Piani et al., 1999). Secreted PPG of *Leishmania mexicana* amastigotes activates the complement system binding to serum mannan-binding proteins, reducing hemolytic activity of normal serum and preventing the opsonization of amastigotes (Peters et al., 1997). *Cryptosporidium parvum* employs the CpMuc4 and CpMuc5 ML-proteins for attachment and invasion of intestinal epithelial cells (Connor et al., 2009). Similarly, highly polymorphic ML-proteins from *Schistosma mansoni* are key factors for the compatibility and interaction of schistosomes with the snail host (Roger et al., 2008).

Recent studies of the mucin-like regions in EBOV and HSV revealed their role in infection. A mouse study of EBOV's mucin-like glycoprotein (Emuc) in virus pathogenesis showed Emuc as a pathogenic factor of EBOV; it causes acute inflammation and tissue injury. In mouse muscle, Emuc induced cell death, and this tissue lesion could be directly mediated by the cytotoxicity of Emuc (Ning et al., 2018). Similarly, the MLR at the N-terminus of HSV-1 surface glycoprotein modulates the HSV-glycosaminoglycan interactions and regulate the affinity, type, and number of glycoproteins involved in the interaction and in the attachment and release of the virus (Delguste et al., 2019).

Many parasitic and viral infections that use MLMs during the infection are life-long, debilitating, and life-threatening diseases (Steverding, 2014; Malvy et al., 2019) with a substantial epidemic potential and need for further research (Malvy et al., 2019). Mucins and MLMs are becoming important markers for diagnostics and targets for drug and vaccine design. MUC1-based structures are used as targets for cancer immunotherapy (Martínez-Sáez et al., 2017) and antibodies against ML-proteins are employed to discriminate *T. cruzi* lineages and to diagnose Chagas disease (Bhattacharyya et al., 2014). However, mucins and MLMs research is still limited by access to pure materials and a poor understanding of the function of these molecules in diseases.

## Production of Mucins and Mucin-Like Molecules and Their Use as Immunomodulators

The physicochemical and biological properties of mucins render them interesting biomarkers for tumor diagnosis (Pett et al., 2017) and models for the production of new biomaterials (Petrou and Crouzier, 2018).

Recombinant protein expression enables the evaluation of mucin structures and their biological role. Human MUC2 structures have been studied using the expression of the C- and N-terminal parts as a recombinant tagged protein in Chinese hamster ovary cells (CHO-K1 cells) (Godl et al., 2002; Lidell et al., 2003). Similarly, the expression of the C-terminal cysteine-rich part of the human MUC5AC mucin in CHO-K1 and a structural analysis, showed that MUC2 and MUC5AC share the sequence (Gly-Asp-Pro-His) for the site of cleavage situated in the GDPH sequence found in the von Wildebrad D4 domain (Lidell and Hansson, 2006). These facts guarantees further progress to study the role of these mucins in human mucus.

To evaluate the role of MUC6 in gastrointestinal cancer; MUC6 was expressed in COS-7, PANC-1, LS 180, and MCF7 cell lines and used in cell invasion and adhesion studies. MUC6 may inhibit tumor cell invasion and slow the development of infiltrating carcinoma (Leir and Harris, 2011). Similarly, the role of MUC5B in pancreatic cancer and respiratory epithelia was assessed by cloning and expression using a mammalian episomal expression vector pCEP-His in 293-EBNA and human lung carcinoma cells (A549) (Ridley et al., 2014). A truncated MUC5AC was employed to assess the interaction of *Helicobacter pylori* with the gastric epithelia using AGS cells. The production of recombinant mucins with diverse structures in different cells is a novel platform to analyze mucin biosynthesis, secretion and functions (Dunne et al., 2017). More recently, larger-scale biomanufacturing of human mucins utilized a codon-scrambling strategy to generate synonymous genes of two mucins of commercial interest in Freestyle 293-F cells. Methods for cDNA design and mucin production in mammalian host production systems were established (Shurer et al., 2019).

The heterogeneity and difficult characterization of isolated glycoproteins together with the need for homogeneous material for drug and vaccine design prompted the chemical synthesis of mucin and MLM related structures. Synthetic antigens induce a strong immune response for diagnostic and vaccine purposes. Mucin glycans from Type-1 core (Pett and Westerlind, 2014) and Type-2 core (Pett et al., 2013) and the combination of synthetic glycans with peptide synthesis by Fmoc-SPPS to obtain core mucin glycopeptides have been reported (Pett et al., 2013; Pett and Westerlind, 2014).

Synthetic tumor-associated mucin glycopeptides have been intensely studied as potential cancer vaccines over the past decade. Cancer cells can be distinguished from normal cells by overexpression of molecular markers on the membrane. Thus, some Tumor-associated carbohydrate antigens (TACAs) are promising targets for the design of anticancer vaccines (Wilson and Danishefsky, 2013; Feng et al., 2016). The MUC1 glycopeptide, which is aberrantly glycosylated and overexpressed in a variety of epithelial cancer has received much attention. MUC1 and Tumor-associated MUC1 are important antigens for tumor vaccines design (Wilson and Danishefsky, 2013) and the induction of MUC1-specific humoral and cellular responses (Martínez-Sáez et al., 2017). High antibody titers were observed for mono- and di-glycosylated glycopeptide vaccine candidates, with sialyl-T_N_ and T_N_ antigens from MUC1 tandem repeats connected to OVA T-cell peptide epitope (Westerlind et al., 2008, 2009). A TA-MUC1 Sialyl-T_N_ glycopeptide (Kaiser et al., 2009) and a fluorinated-substituent analog bearing the Thomsen-Friedenreich antigen also showed a strong and highly specific immune response in mice (Hoffmann-Röder et al., 2010). Recently, a synthetic cancer vaccine candidate consisting of a MUC1 glycopeptide and B-cell epitope was used to break the self-tolerance of the immune system. The glycopeptides were combined with tetanus toxoid as the immune-stimulating carrier to obtain high IgG antibodies titers. A monoclonal antibody generated from the immunization, exclusively bound to tumor-associated MUC1, allowing for the discrimination of human pancreatic cancer (Palitzsch et al., 2016).

Determining the structure of mucin derivatives is important to design specific antigens. Some recent studies in this field include the analysis of the structure of Ser and Thr-linked glycopeptides at an atomic level using X-ray, showing that there is no equivalence of O-glycosylation in Ser and Thr during molecular recognition processes (Martínez-Sáez et al., 2015). A revision of the specificity of cancer-related monoclonal antibodies and a combination of microarray screening and saturation transfer difference STD-NMR also supported the notion that there is specificity for the amino acid (Ser or Thr) in the recognition process (Coelho et al., 2015). Other studies showed that besides the role of the amino acid, the glycosylation in MUC1 peptide strongly affects antibody binding (Movahedin et al., 2017).

Structural studies include the evaluation of a synthetic antitumor vaccine candidate with an unnatural MUC1 α-methylserine in transgenic mice, to show the important role in presentation and dynamics of the sugar moiety displayed by the MUC1 derivative in immune recognition (Martínez-Sáez et al., 2016). In other studies, a library of more than 100 synthetic MUC1 glycopeptides was used to assess the recognition of antibodies induced by three different vaccines, and provided important insights concerning the specificity of anti-glycan antibodies for the design of antitumor vaccines (Pett et al., 2017). Synthetic antitumor vaccine candidates based on mucin glycopeptides and the rational design of cancer vaccines have been reviewed (Gaidzik et al., 2013; Martínez-Sáez et al., 2017).

One of the most studied MLMs are the glycoproteins from *T. cruzi*. The characterization of the glycans and protein core of these molecules, has served as a model to synthesize mucin-like O-glycans, peptides, glycosyl-amino acids, and glycopeptides. Initial synthesis includes the preparation of the O-linked saccharides **1**–**5** (Figure 4) present in *T. cruzi* Colombiana and Tulahuen strains (de Lederkremer and Agusti, 2009). The first synthetic target was disaccharide **1** (Gallo-Rodriguez et al., 1996), which is the basis of synthesizing other molecules including trisaccharides **2** (Gallo-Rodriguez et al., 1998), **3** and **4** (Mendoza et al., 2010), tetrasaccharide **5** (Gallo-Rodriguez et al., 2003), pentasaccharide **6** (Mendoza et al., 2006), and hexasaccharide **7** (Agusti et al., 2015). Further reports include the synthesis of glycan **8** from the *T. cru*zi Y strain (Figure 4) (van Well et al., 2008). Glycosyl amino acids **9** and **10** and disaccharides glycosides **11** and **12** derived from the *T. cruzi* Y strain were synthesized to study the mucins as substrates for *trans*-sialidase activities; i.e., a chemoenzymatic reaction on the glycosyl amino acid **9** was used to obtain the glycopeptide **13**. These studies delivered information about the relaxed acceptor substrate specificity of the *T. cruzi trans*-sialidase, which is important to understand the role of this enzyme during *T. cruzi* infections (Campo et al., 2007).

Further derivatives from *T. cruzi* ML-proteins can be used to discriminate Chagas disease infection for proper diagnostics and treatment. Seven lineage-specific peptides based on the *T. cruzi* trypomastigote small surface antigen (TSSA) with a N-terminal biotinylation, PEG spacer, Gly, and the terminal Cys were synthesized. Analysis of these epitopes showed the potential of synthetic peptides to provide *T. cruzi* antigens and to confirm the disparate geographical distribution in some samples. However, peptides alone were not sufficient to discriminate the strains. But new glycan and glycopeptide epitopes may provide new clinical biomarkers for the prognosis of Chagas disease (Bhattacharyya et al., 2014).

The use of recombinant TSSA and peptides derived from this antigen as a serological marker has been evaluated. Studies done in the last 10 years show detection of specific antibodies in human sera for the diagnosis of Chagas disease (De Marchi et al., 2011), mapping of the antigenic structure, validation of its use as a novel tool for Chagas' disease diagnosis (Balouz et al., 2015), and evaluation of TSSA as an early serological marker of drug efficacy in *T cruzi*-infected children (Balouz et al., 2017). These studies have shown that TSSA is useful as a marker for diagnosis and assessment of treatment efficiency, exhibiting improved sensitivity and specificity.

The interest in antigens from *T. cruzi* MLMs as markers for diagnostics and the development of vaccines has increased over the last years. Recent studies used the trisaccharide derivative **14** containing the immunodominant tGPI-mucin α-Gal epitope from *T. cruzi* to obtain a glycoconjugate with human serum albumin (HSA) as a carrier protein. Mice with an α1,3-galactosyltransferase-knockout, a mouse model for acute Chagas Disease, were immunized with this glycoconjugate and were fully protected from a lethal *T. cruzi* infection (Portillo et al., 2019). Similarly, a conjugate containing the synthetic trisaccharide **15** and BSA was recently introduced as a potential marker for the detection of Chagas disease using serum samples of *T. cruzi*-infected patients (Lopez et al., 2019). Despite these promising results, an effective vaccine against *T. cruzi* infections and a gold standard method for Chagas disease diagnosis are still needed.

## Conclusion and Perspectives

Mucin and mucin-like molecules are important markers and targets for diagnostics and the prognosis of worldwide impact, lifelong, life-threatening, or even potential epidemic diseases such as cancer, Chagas disease, and Ebola Virus infections. There is a link between human mucins, pathogenic mucin-like molecules and their expression in multiple diseases. Changes in mucin and MLM glycosylation is an important factor that modulates molecular recognition by the immune system, differentiation of healthy tumor tissues, and can facilitate infections by pathogens. However, further research is necessary to establish the mechanisms of glycan modifications and other effects of these modifications in the structure and interactions of the glycoproteins.

Diverse challenges remain in using mucin- and MLMs in diagnosis, mucin-based vaccine designs, and the production of mucin-based materials. New strategies for the production of mucins and MLMs through chemical synthesis or expression systems are needed as methods to determine the properties of these molecules. It is also necessary to find methods for easy determination, characterization, and quantification of mucin glycosylation in normal and abnormal tissues. We require further analysis of mucin like molecules from pathogens to understand the interaction of these molecules with human receptors, and to determine how MLMs support the evasion of pathogens from the immune system. In addition, future research should also include the synthesis of new epitopes to provide new clinical biomarkers for diagnostics and the development of new antigens for the design of cancer vaccines.

## Author Contributions

SP and DV wrote the review. DV and PS revised the manuscript.

### Conflict of Interest

The authors declare that the research was conducted in the absence of any commercial or financial relationships that could be construed as a potential conflict of interest.
