# Corrigendum: Mucins and Pathogenic Mucin-Like Molecules Are Immunomodulators During Infection and Targets for Diagnostics and Vaccines

**DOI:** 10.3389/fchem.2019.00846

**Published:** 2019-12-05

**Authors:** Sandra Pinzón Martín, Peter H. Seeberger, Daniel Varón Silva

**Affiliations:** ^1^Department of Biomolecular Systems, Max Planck Institute of Colloids and Interfaces, Potsdam, Germany; ^2^Department of Biology, Chemistry and Pharmacy, Freie Universität Berlin, Berlin, Germany

**Keywords:** mucins, mucin-like molecules, O-glycoproteins, cancer, parasites, virus, infection

In the original article, there was a mistake in Figure 4 as published. The strain for three structures was incorrectly assigned in the figure legend. Structures 3 and 6 were corrected and structures 6–8 required renumbering to fit the text. The correct Figure 4 and legend appear below.

**Figure 4:**
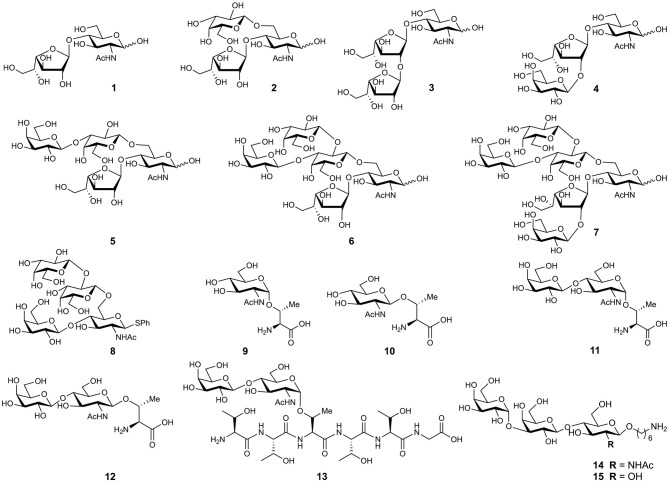
Chemical structure of synthesized glycans from MLMs of *Trypanosoma cruzi* Colombiana strain (**1**, **2**, **6**, **7**), Tulahuen strain (**3**–**5**), and Y strain (**8**–**13**).

The authors apologize for this error and state that this does not change the scientific conclusions of the article in any way. The original article has been updated.

